# Evaluation of dental practitioners’ knowledge and attitudes regarding drug interactions: a cross-sectional survey

**DOI:** 10.1186/s12903-026-07832-7

**Published:** 2026-02-10

**Authors:** Seyhan Karaaslan, Gülin Acar, Salih Eren Meral

**Affiliations:** 1 Republic of Turkey Ministry of Health, Ankara Bilkent City Hospital, Ankara, 06800 Turkey; 2 Faculty of Dentistry, Department of Oral and Maxillofacial Surgery, Kırıkkale University, Kırıkkale, Turkey; 3 Faculty of Dentistry, Department of Oral and Maxillofacial Surgery, Hacettepe University, Ankara, Turkey

**Keywords:** Dental practitioners, Drug information sources, Questionnaire study, Drug–drug interactions

## Abstract

**Objectives:**

In dentistry, various medications are commonly used to manage conditions and maintain patient comfort. The use of multiple medications at the same time, known as polypharmacy, often occurs to manage multiple conditions. Drug interactions in dentistry present considerable risks but reveal gaps in dentists’ knowledge. Recognizing drug interaction prevalence is essential for better prescribing and patient outcomes. This investigation aims to evaluate dentists’ comprehension of pharmaceutical interactions, clarify common misconceptions, and examine the impact of years of professional experience, specialty/doctoral training, and dental specialty field, and analyze the factors influencing dental professionals’ prescribing behaviors.

**Methods:**

This study engaged 217 dental practitioners (dental interns and post-graduate dentists) from diverse specialties in Turkey. Participants completed a 23-question survey on drug interactions, medication management, and demographic data were analyzed descriptively to outline participant responses. Chi-square and Fisher’s exact tests compared responses according to professional experience, specialty, and education.

**Results:**

Of 217 dentists surveyed, 73.2% had 0–10 years of experience and 76% worked in public hospitals or university clinics. Nearly half (46.5%) had specialized or doctoral training. About 64.5% reported limited knowledge of drug mechanisms, and 71.9% saw their ability to manage drug interactions as inadequate. Less than a third identified clarithromycin as a CYP3A4 inhibitor, and over 60% couldn’t name key macrolide interactions. Only 29.2% recognized drugs that raise INR with warfarin, with 41.9% lacking this knowledge. Correct responses varied by specialty, with oral and maxillofacial surgeons performing best. Nearly all (94.9%) would use decision support tools, and 44.7% wanted more training on drug interactions.

**Conclusions:**

The study highlights the importance of specialized educational initiatives and clinical decision support systems in enhancing dentists’ understanding and management of drug interactions. Addressing these knowledge gaps is crucial for improving patient safety and optimizing pharmacotherapy within dental care settings.

**Supplementary Information:**

The online version contains supplementary material available at 10.1186/s12903-026-07832-7.

## **Background**

Medications remain one of the most common treatments in dental healthcare, but they also serve as a significant source of iatrogenic morbidity and mortality [1]. In dentistry, to manage patient comfort and treat oral health problems, dentists routinely prescribe a variety of pharmacological agents to meet these needs effectively. Among the most utilized medications are anti-inflammatory agents, analgesics, antibiotics, and local anesthetics [2–6].

Polypharmacy, often seen in elderly individuals, increases the risks of side effects and drug interactions [7]. As it increases, so do adverse drug interactions in dentistry [8], leading to potential drug–drug interactions (DDI) in dental practice [8]. While dental medication courses are typically shorter in duration than those in general medicine, interactions between dental drugs and patients’ current medications can still carry substantial risks [9]. Additionally, understanding the therapeutic effects and potential adverse reactions is essential for safe prescribing practices [10].

This study aims to evaluate dentists’ knowledge of pharmaceutical interactions, explain the most important points or concepts, and determine factors influencing their prescribing habits. It addresses gaps in existing research, as few studies have thoroughly evaluated dentists’ knowledge and practices concerning drug interactions, especially regarding frequently prescribed antibiotics and high-risk medications in dentistry.

## **Methods**

Prior to conducting this survey, approval was obtained from the Clinical Research Ethics Board at Hacettepe University (Clinical Trial Number: SBA 25/234), ensuring that the study conformed to ethical standards and safeguarded the rights and well-being of the participants. This study employed an observational design to assess the knowledge and skills of dentists and postgraduate doctors or specialists concerning drug-drug interactions. Data collection was conducted via a cross-sectional approach through the administration of online questionnaires between March 2025 and July 2025, with approval from the university’s Dean and the survey was open for six months. Before participation, all individuals received a concise briefing on the study’s objectives, and informed consent was secured from each participant.

The data were collected using a questionnaire via Google Forms, and responses were exported to Google Sheets, reducing entry errors. The questionnaire included multiple-choice and open-ended questions (Q), distributed voluntarily to participants on a voluntary basis.

### Inclusion criteria

All active dental practitioners (including interns and post-graduate specialists) currently practicing in Turkey who voluntarily agreed to participate in the study.

### Exclusion criteria

- Participants who did not provide informed consent.
- Questionnaires with more than 20% missing data or incomplete sections regarding primary pharmacological knowledge.
- Duplicate submissions from the same participant.
- Retired dentists or those not actively engaged in clinical practice (not prescribing medications).
- Responses containing evident logical contradictions or uniform answering patterns (e.g., selecting the same option for all items).

Participants were chosen from public hospitals, universities, private practices, or dental hospitals. The selection process was managed by the Personnel and Student Affairs Directorate.

### Instrument development and validation

The study consisted of a 23-question structured questionnaire. The choice to target specific drug groups—mainly antibiotics (such as macrolides, metronidazole), anticoagulants (like warfarin), and NSAIDs—was driven by their frequent prescription in dental surgery and their potential to cause severe iatrogenic complications. These medication categories are among the most used in dentistry and have significant risks of clinical issues via critical pathways, including CYP3A4 inhibition, INR increase, and interactions with antihypertensive drugs.

#### Validation involved 2 phases

##### Expert Review: 

A panel of 4 specialists, including surgeons and a clinical pharmacist, assessed items for content validity.

##### Pilot Testing: 

An initial test with 20 dentists confirmed face validity by ensuring clear terminology and ease of response.

The questionnaire used in this study contained twenty-three questions (Study Questionnaire) specifically designed for participants. The first four questions gathered demographic information. The remaining questions assessed participants’ knowledge levels, competencies, clinical practices, and methods for accessing information about drug interactions.

Participants’ responses regarding their knowledge and clinical practices were analyzed using frequency and percentage distributions. Demographic data and survey answers were presented as descriptive statistics with frequency (*N*) and percentage (%). The chi-square test compared correct and incorrect answers based on experience, training, and specialty, with Fisher’s exact test used if chi-square assumptions were unmet. Significance was set at *p* < 0.05.

### Statistical power analysis and sample size

To indicate the effect size in these tests, Cohen’s *w* coefficient was used. The *w* value is a standardized measure that expresses the extent to which observed frequencies deviate from expected frequencies and allows interpretation of the magnitude of differences between groups [11]. According to the classification made by Cohen, a *w* value of 0.10 represents a small effect size, 0.30 represents a medium effect size, and 0.50 represents a large effect size [12]. In the power analysis (version 3.1.9.7) for the chi-square test, calculations used effect size (w) = 0.30, significance level α = 0.05, power (1–β) = 0.95, and df = 3 (for four experience categories: 0–5, 6–10, 11–20, 20+ years). A total of 217 valid responses were analyzed, which exceeded the minimum required threshold for high-confidence statistical inference.

## **Results**

A total of 217 dentists participated in the study. The majority had 6–10 years (37.3%) or 0–5 years (35.9%) of clinical experience. Nearly half of the respondents (46.5%) had completed a specialty or doctoral training, while 15.2% were currently enrolled in such programs. General dentistry (38.2%) and oral and maxillofacial surgery (24.4%) were the most represented specialties. Most participants were working in public hospitals (40.1%) or university clinics (35.5%) (Table 1).

**Table 1:** Demographic information

Demographic information		*N*	%
Professional experience	0–5 y	78	35.9
	6–10 y	81	37.3
	11–20 y	33	15.2
	20 y and +	25	11.5
Education Level	Yes, specialty/PhD Completed	101	46.5
	No Specialized Training	83	38.2
	Ongoing Specialty Education	33	15.2
Specialty or PhD Field*	General Dentistry	83	38.2
	Oral and Maxillofacial Surgery	53	24.4
	Other Specialties (Endo, Perio, etc.)	81	37.4
Workplace	Public Hospital	87	40,1
	University Hospital	81	36,9
	Private Clinic	45	21,0
	Other	4	2N

*N* frequency, *%* percent, *y* years

“Specialty/PhD Completed” excludes those with ongoing education

### Analysis of pharmacological knowledge

When asked about drug groups with significant potential interactions during dental treatments, 44.2% of participants selected “all of them,” while 22.1% mentioned anticoagulants specifically. Regarding their familiarity with drug mechanisms, 64.5% stated partial knowledge, 27.2% reported adequate knowledge, and 8.3% were unfamiliar (Table 2).

**Table 2:** Accuracy of pharmacological knowledge regarding drug interactions

Questions	Answers	*N*	%
General Interaction Risk in Dentistry(Q5)	Anticoagulants	48	22.1
	Antibiotics	12	5.5
	Antidepressants	7	3.2
	Corticosteroids	17	7.8
	Beta blockers	18	8.3
	All	96	44.2
	None	19	8.8
Mechanism Familiarity Drug (Q7)	Yes	59	27.2
	No	18	8.3
	Partially	140	64.5
Enzyme inhibition of the CYP3A4 causes drug interactions? (Q12)	Clarithromycin	63	29.0
	Azithromycin	19	8.8
	Metronidazole	29	13.4
	I don’t know	67	30.9
	All of the macrolid antibiotics	11	5.1
	Statins	31	14.3
Macrolide group of interaction risks (cause serious side effects) (multiple options can be selected) (Q13)	Antiarrhythmics	24	11.1
	Benzodiazepines	37	17.1
	SSRIs (e.g., citalopram)	28	12.9
	Calcium channel blockers	48	22.1
	Fluoroquinolones (e.g., levofloxacin, moxifloxacin)	18	8.3
	None	3	1.4
	No knowledge	132	60.8
	Erythromycin	32	14.7
Colchicine Toxicity Risk (Q14)	Clarithromycin	91	41.9
Disulfiram-like Reaction in the usage of Metronidazole (Q16)	Caffeine	9	4.1
	No knowledge	25	11.5
	Beta blockers	15	6.9
	Alcohol	156	71.9
	Aspirin	12	5.5
	No knowledge	50	23.0
	Antibiotics	25	11.5
NSAID Adverse Effects (Q17)	Beta-blokers	21	9.7
	ACE inhibitors	108	49.8
	SSRIs	13	6.0
	Clarithromycin	52	24.0
	Amoxicillin	35	16.1
Warfarin INR Potentiation- most commonly interacts with warfarin (Q18)	Paracetamol	63	29.0
	Azithromycin	35	16.1
	No knowledge	32	14.7
	Clarithromycin	52	24.0
	Amoxicillin	35	16.1

*Q *question, *N *frequency, *%* percent

In clinical practice, the drug group most frequently considered for interactions was anticoagulants (49.3%), followed by antibiotics (34.1%) and corticosteroids (26.3%) (Q6). Participants primarily obtained information about patients’ medications by questioning medical history (61.3%) and requesting medication lists (51.6%). Electronic health records were used by 34.6%.

### Professional attitudes and perceived competence

The study identifies a strong professional readiness for clinical support tools. A significant majority (71.9%) explicitly rated their DDI management competence as “inadequate,” reflecting a high degree of self-awareness. This perceived inadequacy is directly linked to the overwhelming demand for Clinical Decision Support Systems (CDSS) (Table 3). Perception of competence in managing drug interactions is also low. Most participants rated themselves as “inadequate” (71.9%; *N* = 156). A total of 94.9% (*N* = 206) said they would use a medication decision support system when prescribing medications. Only 2.3% (*N* = 5) answered “no,” while 2.8% (*N* = 6) said “undecided.” This result indicates a high level of acceptance of the need for clinical decision support systems (Table 3).

**Table 3:** Professional attitudes, competencies, and methods for obtaining patient medication information

Questions	Answers	*N*	%
Anamnesis Consistency (Q8)	Yes, in every patient	179	82.5
	Mostly/Sometimes	38	17.5
Self-Rated Warfarin Knowledge (Q19)	Strong knowledge	13	6.0
	Little to No knowledge	151	69.5
Overall DDI Competence (Q20)	Good knowledge	13	6.0
	Inadequate	156	71.9
	Moderate	47	21.7
	Adequate	14	6.5
Preferred Training Topics (Q21)	Specific drug interactions	97	44.7
	Patient safety in drug use	48	22.1
	Rational drug use and digital tools	72	33.2
Decision Tool Interest (DDI) (Q22)	Yes	206	94.9
	No	5	2.3
	Undecided	6	2.8
Medication Info Sources Access(Q 23)	E-prescription	61	28.1
	TITCK official website	95	43.8
	Vademecum	49	22.6
	Colleagues	12	5.5

*Q *Question, *N *frequency, *% *percent

Finally, when asked which source to use when seeking information about drug interactions, most participants chose the Official Website of the Turkish Medicines and Medical Devices Agency (TİTCKRS) (43.8%; *N* = 95). This was followed by the e-prescription system (28.1%; *N* = 61) and Vademecum (modern medicine guide) (22.6%; *N* = 49). The percentage of those who preferred their colleagues was quite low (5.5%; *N* = 12) (Table 3).

### Comparison of the accuracy of the answers given according to years of professional experience

Based on the chi-square analyses, a statistically significant relationship was found between years of professional experience and responses to question 13 (χ² = 14.92; *p* = 0.002). For all remaining questions, the *p*-values exceeded the threshold for statistical significance. Consequently, no meaningful association was identified between years of professional experience and correct response rates (*p* > 0.05). The finding of a significant correlation between years of professional experience and knowledge level only for question 13 suggests that knowledge of drug interactions, particularly with macrolide antibiotics, strengthens with clinical experience. No notable differences for the remaining questions suggest that professional expertise alone does not account for drug interaction knowledge (Table 4).

**Table 4:** Participants’ perceived competencies and methods of accessing information regarding drug interactions

Questions		Years of professional experience								*X* ^*2*^	*p*
		0–5 y		6–10 y		11–20 y		20 y +			
		*N*	%	*N*	%	*N*	%	*N*	%		
Q5	True	23	47,9	12	25,0	8	16,7	5	10,4	5,118	0,163
	False	55	32,5	69	40,8	25	14,8	20	11,8		
Q12	True	18	28,6	28	44,4	9	14,3	8	12,7	2,704	0,440
	False	60	39,0	53	34,4	24	15,6	17	11,0		
Q13	True	45	17,7	52	20,5	76	29,9	81	31,9	14,92	0,002**
	False	21	31,3	22	32,8	10	14,9	14	20,9		
Q14	True	12	15,4	7	8,6	3	9,1	1	4,0	3,441	0,329
	False	66	84,6	74	91,4	30	90,9	24	96,0		
Q16	True	52	66,7	60	74,1	25	75,8	19	76,0	1,697	0,637
	False	26	33,3	21	25,9	8	24,2	6	24,0		
Q17	True	37	47,4	43	53,1	13	39,4	15	60,0	2,994	0,397
	False	41	52,6	38	46,9	20	60,6	10	40,0		
Q18	True	16	20,5	31	38,3	8	24,2	8	32,0	6,578	0,087
	False	62	79,5	50	61,7	25	75,8	17	68,0		

*N *frequency, *% *percent, *Q* Question, *y* years

### Comparison of correct answers to information-related questions according to specialization/doctorate education in dentistry

The results of the chi-square analysis comparing the correct answers given to information-related questions according to dental specialty/doctoral training status are shown in Table 6. Dental specialty or doctoral training significantly affected correct answer rates for certain questions (*p* < 0.05). Chi-square analysis showed a significant association between this variable and correct responses to questions 13, 17, and 18, while no significant group differences were found for other questions (*p* > 0.05) (Table 5).

**Table 5:** Comparison of correct answers to information-related questions according to specialization/doctorate education in dentistry

Interaction Risk Area	Specialty/PhD completed (%)	No specialized Training (%)	*p*-value
Macrolide Interaction Groups (Q13)	62.6	21.7	0.01**
NSAID-ACEi Interactions (Q17)	78.0	26.5	0.043*
Warfarin Potentiation (Q18)	50.6	13.3	0.032*

*N* frequency, *%* percent, *Q *Question, *y* years

*N refers to the number of responses for each answer option. If a question allowed multiple selections, *N* may exceed the total number of participants. For single-choice questions, *N* represents the number of participants selecting each option

Table 6 shows the results of chi-square analyses comparing correct answers to Q5, Q12, Q13, Q14, Q16, Q17, and Q18 across dental specialties.

For Q5, the field of expertise was significantly linked to responses (χ² = 17.387; *p* = 0.018). The highest correct response rate was seen in oral and maxillofacial surgeons at 59.5%, while the incorrect response rate in this group was 40.5%.

Responses to Q12 also showed significant differences across specialties (χ² = 14.067; *p* = 0.033). The highest correct response rate was in the oral and maxillofacial surgery group, at 61.2%, and the incorrect response rate in this area was 38.8%.

In the analysis for Q14, differences in knowledge about antibiotics that increase the risk of colchicine toxicity were observed by specialty, although this difference was not significant (χ² = 6.403; *p* = 0.515). The groups with the highest correct response rates were general dentistry (34.8%), oral and maxillofacial surgery (26.1%), and endodontics (21.7%). Correct response rates were quite low in other specialties, and some (pediatric dentistry, prosthodontics, oral diagnosis, and radiology) did not provide any correct responses at all. This suggests that knowledge of the pharmacokinetics of colchicine is generally limited and does not differ significantly by specialty.

In Q16, knowledge about the interaction between metronidazole and alcohol varied by specialty, with the highest correct response rates in general dentistry (35.3%) and oral and maxillofacial surgery (27.6%). Thus, differences in knowledge by specialty were not statistically significant (Table 6).

According to the chi-square analyses in Q17 and Q18, there was no statistically significant relationship between specialty and correct response rates for either question (χ² = 9.075; *p* = 0.336 for Q17; χ² = 13.292; *p* = 0.084 for Q18) (Table 6).

**Table 6:** Percentage of correct responses by dental specialty

Knowledge Questions (Correct %)	Gen. Dent. (*N* = 83)	OMFS (*N* = 53)	Endo (*N* = 20)	Perio (*N* = 13)	Others* (*N* = 48)	*p*-value
Q5 (Gen. Interaction)	18.9	59.5	21.1	4.8	11.5	0.018*
Q12 (CYP3A4 Inhibition)	18.2	61.2	25.0	20.0	14.7	0.033*
Q13 (Macrolide Risks)	80.0	91.0	68.0	76.0	64.3	0.044*
Q14 (Colchicine Toxicity)	34.8	26.1	21.7	8.7	8.7	0.515
Q16 (Metronid./Alcohol)	35.3	27.6	8.3	7.7	21.1	0.067
Q17 (NSAID-ACEi)	36.1	28.7	12.0	7.4	15.8	0.336
Q18 (Warfarin Potent.)	31.7	34.9	9.5	9.5	14.4	0.084

*Includes: Pediatric Dentistry, Orthodontics, Prosthodontics, Oral Diagnosis/Radiology, and Restorative Treatment

*Gen. Dent.* General Dentistry, *OMFS* Maxillofacial Surgery, *Endo* Endodontics, *Perio* Periodontology

## **Discussion**

In pharmacotherapy, medication interactions are crucial, especially in polypharmacy, where patients often take multiple medications. This is especially important in dentistry, where professionals see patients with chronic conditions needing various treatments. Despite the prevalence of these cases, few studies have thoroughly assessed dentists’ ability to recognize and manage drug interactions, particularly those related to antibiotics and frequently prescribed medications [13]. This study evaluates dentists’ knowledge and readiness concerning drug interactions in dental practice. The risk of adverse drug reactions rises significantly with the number of medications, making prediction and management more challenging [13, 14]. The survey indicates substantial deficiencies in dentists’ pharmacological knowledge, particularly about DDI involving antibiotics. Participants were selected from various dental specialties and levels of experience, increasing the diversity of the findings within the Turkish dental community. This study identified knowledge gaps and dentists’ openness to interventions such as decision-support systems, offering valuable insights for educational and technological improvements.

Recent studies show dentists often face a gap between their practices and pharmacological knowledge, reflected in our findings. Dental students often make prescription errors and overuse antibiotics due to limited knowledge of drug interactions [15]. Sippli et al. found many dentists are unaware of drug safety concerns, such as anticoagulant interactions, and lack confidence in prescribing analgesics [16]. Similarly, our study shows that while 82.5% collected medication histories, only 6.5% felt competent managing drug interactions, demonstrating a gap between knowledge and practice.

The lack of awareness about macrolide interactions is particularly concerning in settings where multiple drugs are used together [15]. Combining macrolides with statins, anticoagulants, or immunosuppressants can cause serious side effects like bleeding complications, or kidney damage [16]. Since macrolides are commonly chosen for patients allergic to penicillin, this knowledge gap could lead to significant clinical harm if dentists are unaware of these risky drug combinations [7]. Macrolide antibiotics are commonly prescribed for similar conditions. Clarithromycin inhibits CYP3A4, so taking it with a CYP3A4-metabolized calcium channel blocker can increase the blocker’s effect. Azithromycin is a weak inhibitor of CYP3A4 and does not have this concern. The kidney is vulnerable to poor perfusion, making acute kidney injury a significant risk when macrolide antibiotics interact with calcium-channel blockers. This likely results from hemodynamic changes, as evidenced by an increased risk of hospitalization due to hypotension in this context [19, 20]. Less than one-third of the participants accurately recognized clarithromycin as an inhibitor of CYP3A4, and more than 60% failed to identify any significant interactions between macrolides and other classes of medication. These findings suggest that knowledge of macrolide drug interactions is limited in some specialties and that this awareness may be higher, particularly in specialties that frequently encounter surgical interventions. A general assessment of the results revealed that correct response rates for both questions were significantly higher in oral and maxillofacial surgery, while incorrect response rates were generally above 75% in other specialties. These results suggest significant differences in knowledge about drug interactions across specialties, depending on clinical practice.

Warfarin is the most widely used oral anticoagulant worldwide, effective in preventing thromboembolic events in patients with cardiovascular risk factors [17]. Being a drug with a narrow therapeutic range makes it susceptible to drug interactions with antibiotics used in dental treatments [18]. Various classes of antibiotics, encompassing penicillin derivatives, fluoroquinolones, cephalosporins, sulfonamides, macrolides, and metronidazole, have been identified as agents that can enhance the anticoagulant effects of warfarin, consequently resulting in an elevated international normalized ratio (INR) and a heightened risk of hemorrhage [19–21]. Metronidazole, another antibiotic commonly used in dentistry, also potentiates warfarin, elevating the INR to supratherapeutic levels in a considerable proportion of patients [11]. According to our findings, a significant proportion of participants demonstrated limited knowledge regarding these high-risk interactions. Only 29.2% could identify drugs that raise INR with warfarin, while 41.9% were unaware of any warfarin interactions. These findings underscore a significant deficiency in educational understanding, especially considering the prevalent administration of antibiotics, including macrolides and metronidazole, within the realm of dental practice. Our results show low awareness of warfarin–antibiotic interactions, consistent with earlier surveys. Our findings align with international data. In the United Arab Emirates, only 5–6% of dentists knew about common antithrombotic drugs, despite often seeing such patients [22]. A recent cross-sectional study, involving 153 dentists, was conducted by Erçal et al. The study revealed that, despite having adequate knowledge of ASA and warfarin, only 16% of dentists could identify serious interactions between metronidazole or macrolides and warfarin’s INR effects. These antibiotics are often used in dental procedures. Without understanding these interactions, clinicians may unintentionally cause preventable adverse drug events in vulnerable patients [23].

Our results highlight a significant deficiency between knowledge and practice in dental pharmacology. While 82.5% of participants routinely obtained patients’ medication histories, only 6.5% felt confident managing DDI, suggesting that procedural compliance does not always ensure safe prescribing. Fewer than one-third of dentists identified clarithromycin as a CYP3A4 inhibitor, and over 60% failed to recognize relevant macrolide interactions. Likewise, only 29.2% identified drugs that could increase INR when combined with warfarin, and 41.9% reported no knowledge of warfarin interactions. These results reveal a lack of educational information in pharmacology with serious implications for patient safety, especially in cases of polypharmacy.

Moreover, insufficient comprehension of these interactions may adversely affect patient care, particularly among vulnerable populations. It is essential for dental professionals to maintain current knowledge of pharmacological guidelines to reduce the risk of detrimental drug interactions. Ongoing professional development and interdisciplinary case evaluations can enhance awareness of clinically significant DDIs and promote safer prescribing practices to improve patient outcomes [24]. Regular training sessions focused on drug interactions and their implications in dental care are crucial for enhancing dentists’ competence in managing complex medication regimens [9].

Similar surveys by Erçal et al. and Gaballah et al. also show low awareness among dentists regarding antithrombotic and antibiotic interactions, indicating a widespread issue that calls for continuing education and the use of clinical decision-support tools in dental practice [22, 23].

Our survey found differences in drug interaction knowledge among dental specialties and training levels. Dentists with advanced training, particularly in oral and maxillofacial surgery, performed better on interaction questions, suggesting their higher pharmacological proficiency. Literature also shows that structured continuing education boosts dentists’ confidence and safety in prescribing and recognizing drug interactions [23]. This finding aligns with Bakir et al., who found only 64.2% of general dentists felt adequately informed about drug interactions—the lowest among pharmacologic topics—despite nearly 98% feeling comfortable with drug indications [25]. Antibiotics knowledge did not significantly increase 10with experience among students. Their survey and focus groups showed dentists often rely on anecdotal evidence or defer to physicians for medication management and lack access to current drug information [26]. This causes uncertainty and inconsistent DDI management, emphasizing the need for better education. Respondents were uneasy about managing drug interactions, highlighting persistent knowledge gaps and the need for continued professional development.

Encouragingly, many dentists show a strong desire for updated clinical guidance and decision-support tools. In our survey, 44.7% of participants requested more education on drug interactions and 33.2% on rational prescribing and digital tools. This proactive approach highlights a growing recognition of the challenges in pharmacotherapy and a willingness to improve the knowledge gap. Almost all respondents (94.9%) are willing to use drug-decision support tools, showing strong motivation to overcome knowledge gaps. Similar patterns are seen internationally. Sippli et al. emphasized that integrating digital clinical decision support systems (CDSS) into daily dental practice could enhance safety and reduce errors, especially when managing medically complex patients [27]. Schneider-Smith et al. note that dental providers are interested in antibiotic stewardship. Pilot studies of the “Drugs4Dent” digital decision support system showed it was well accepted, user-friendly, and may enhance safety in dental practice [28]. Dentists participating in that pilot found the system to be intuitive and valuable, concurred that it enhanced their ability to prescribe in accordance with best-practice guidelines, and addressed critical knowledge gaps not covered by existing resources [28]. This tool, now a commercial product, demonstrates how decision support can benefit dental practices. These results suggest that closing the knowledge gap needs a comprehensive strategy, such as improved pharmacological education, integrating clinical decision support systems into operations, and encouraging interdisciplinary collaboration. Indeed, recent reviews advocate for continuous professional education and the involvement of tools such as pharmacists’ consultations and electronic interaction checkers to improve medication safety in dentistry [14]. By implementing these strategies, dental professionals can boost their confidence in handling drug interactions and ultimately improve patient safety in a time marked by increasing polypharmacy.

The study provides valuable insights into dentists’ perspectives on drug interactions; however, its cross-sectional design limits the ability to assess changes in attitudes over time. Using a voluntary sample from Turkish institutions raises questions about wider applicability. Focusing only on certain drug groups in the questionnaire may overlook other important interactions, limiting data comprehensiveness. Future research should use longitudinal designs and broader sampling to enhance understanding in dental pharmacology.

### Methodological limitations

While this study’s cross-sectional design provides a preliminary analysis, it does not establish causality. Furthermore, participation bias may exist, as those with a greater interest in pharmacology are more likely to respond. Additionally, the sample is predominantly institutional, potentially overlooking the unique challenges faced by independent private physicians.

## Conclusion

In summary, this study demonstrates that while dental practitioners recognize the importance of drug interactions in clinical practice, significant gaps remain in their pharmacological knowledge and confidence in managing these risks. The findings highlight that educational background and specialty influence awareness, but overall, there is a clear need for targeted training and practical support. These results reinforce the study’s aim: to identify knowledge deficits and attitudes toward drug interactions among dentists, and to emphasize the value of ongoing education and decision-support systems in promoting safer prescribing practices and improving patient outcomes.

## Supplementary Information

Supplementary Material 1.

## Data Availability

Yes. The datasets used and/or analyses during the current study are available from the corresponding author on reasonable request.

## References

[1] Fung KW, Kapusnik-Uner J, Cunningham J, Higby-Baker S, Bodenreider O. Comparison of three commercial knowledge bases for detection of drug-drug interactions in clinical decision support. J Am Med Inform Assoc. 2017;24(4):806–12. 28339701 10.1093/jamia/ocx010 PMC6080681

[2] Hargreaves K, Abbott P. Drugs for pain management in dentistry. Aust Dent J. 2005;50:S14–22. 16416713 10.1111/j.1834-7819.2005.tb00378.x

[3] Shenoy S, Pasha Z, Rajendiran S, Ahmed SO, Kant K, Shetti AN. The intersection of Pharmacology and dentistry: a comprehensive overview of pharmacotherapeutics. Int J Pharm Chem Anal. 2023;10:66–9.

[4] Cope AL, Chestnutt IG. Inappropriate prescribing of antibiotics in primary dental care: reasons and resolutions. Prim Dent J. 2014;3(4):33–7. 25668373 10.1308/205016814813877333

[5] Badrov M, Marovic D, Tadin A. Antibiotics knowledge and prescription patterns among dental practitioners in Croatia, Bosnia and Herzegovina, and Serbia: a comparative e-survey with a focus on medically healthy and compromised patients. Antibiotics. 2024;13(11):1061. 39596755 10.3390/antibiotics13111061 PMC11591130

[6] Oberoi SS, Dhingra C, Sharma G, Sardana D. Antibiotics in dental practice: how justified are we. Int Dent J. 2015;65(1):4–10. 25510967 10.1111/idj.12146 PMC9376535

[7] Maher RL, Hanlon J, Hajjar ER. Clinical consequences of polypharmacy in elderly. Exp Opin Drug Saf. 2014;13(1):57–65. 10.1517/14740338.2013.827660 PMC3864987 24073682

[8] Hersh EV, Moore PA. Drug interactions in dentistry: the importance of knowing your cyps. J Am Dent Association. 2004;135(3):298–311. 10.14219/jada.archive.2004.0178 15058617

[9] Sharma S, Amit, Sharma K, Neemawat K, Sharma L, Pilania D. Concurrent prescribing: evaluation of its knowledge among dentists. Natl J Maxillofacial Surg. 2019;10(1):73–7. 10.4103/njms.NJMS_21_18 PMC6563645 31205392

[10] Kaur S, Singh T, Kainth A, Kaur A, Kainth M, Bansal S. Drugs and dentistry: a review. J Dent Panacea. 2023;5(4):142–8.

[11] Büyüköztürk Ş. Sosyal Bilimler İçin Veri Analizi El Kitabı 23. baskı, Pegem Akademi Yayıncılık. 2018.

[12] Cohen, J. Statistical Power Analysis for the Behavioral Sciences 2nd edn. Routledge; 1988.

[13] Colibășanu D, Ardelean SM, Goldiș F-D, Drăgoi M-M, Vasii S-O, Maksimović T, Colibășanu Ș, Șoica C, Udrescu L. Unveiling Drug-Drug interactions in dental patients: a retrospective real-world study. Dentistry J. 2025;13(6):255. 10.3390/dj13060255 PMC12191789 40559158

[14] De Oliveira MLR, Nery GO, Torresan TT, Arcanjo RA, Ferreira MBC, Montagner F. Frequency and characterization of potential drug interactions in dentistry—a cross-sectional study. Clin Oral Invest. 2022;26(11):6829–37. 10.1007/s00784-022-04644-1 35930141

[15] Piacentini N, Trifiró G, Tari M, Moretti S, group U, Arcoraci V. Statin–macrolide interaction risk: a population-based study throughout a general practice database. Eur J Clin Pharmacol. 2005;61(8):615–20. 16044339 10.1007/s00228-005-0972-z

[16] Wu Y, Bi W-T, Qu L-P, Fan J, Kong X-J, Ji C-C, Chen X-M, Yao F-J, Liu L-J, Cheng Y-J. Administration of macrolide antibiotics increases cardiovascular risk. Front Cardiovasc Med. 2023;10:1117254. 36910529 10.3389/fcvm.2023.1117254 PMC9996752

[17] Holbrook AM, Pereira JA, Labiris R, McDonald H, Douketis JD, Crowther M, Wells PS. Systematic overview of warfarin and its drug and food interactions. Arch Intern Med. 2005;165(10):1095–106. 15911722 10.1001/archinte.165.10.1095

[18] Lane MA, Zeringue A, McDonald JR. Serious bleeding events due to warfarin and antibiotic co-prescription in a cohort of veterans. Am J Med. 2014;127(7):657–63. e652. 24657899 10.1016/j.amjmed.2014.01.044 PMC4116816

[19] Vega AJ, Smith C, Matejowsky HG, Thornhill KJ, Borne GE, Mosieri CN, Shekoohi S, Cornett EM, Kaye AD. Warfarin and antibiotics: drug interactions and clinical considerations. Life. 2023;13(8):1661. 37629518 10.3390/life13081661 PMC10455514

[20] Patel S, Preuss CV. Warfarin. in StatPearls [Internet]. eds. Abai, B. et al. StatPearls Publishing; 2024.

[21] Baillargeon J, Holmes HM, Lin Y-L, Raji MA, Sharma G, Kuo Y-F. Concurrent use of warfarin and antibiotics and the risk of bleeding in older adults. Am J Med. 2012;125(2):183–9. 22269622 10.1016/j.amjmed.2011.08.014 PMC3712345

[22] Gaballah K, Hassan M. Knowledge and practice of dentists managing patients on antithrombotic medications: a cross-sectional survey. Eur J Dentistry. 2022;16(04):775–80. 10.1055/s-0041-1739436 PMC9683875 35016232

[23] Erçal P, et al. Knowledge and practices of dentists toward patients on antithrombotic medications: a cross-sectional survey. Hosp. Pract. 2023;51:12–21.

[24] Hersh EV, Moore PA. Adverse drug interactions in dentistry. Periodontol. 2008;46:109–42. 10.1111/j.1600-0757.2008.00224.x 18201348

[25] Bakır Ş, Bakır EP, Polat G, Temizyürek S. Attitudes and behaviors of dentists regarding rational drug use. J Dent Sci Educ. 2024;2(3):70–80.

[26] Schneider-Smith EG, Suda KJ, Lew D, Rowan S, Hanna D, Bach T, Shimpi N, Foraker RE, Durkin MJ. How decisions are made: antibiotic stewardship in dentistry. Infect Control Hosp Epidemiol. 2023;44(11):1731–6. 37553682 10.1017/ice.2023.173 PMC10782556

[27] Sippli K, Rieger MA, Huettig F. GPs’ and dentists’ experiences and expectations of interprofessional collaboration: findings from a qualitative study in Germany. BMC Health Serv Res. 2017;17(1):179. 28270205 10.1186/s12913-017-2116-4 PMC5341464

[28] Teoh L, Biezen R, Taylor M, Sinnott RO, McCullough MJ. Acceptability and usability of Drugs4dent^®^, a dental medicines decision tool–a pilot study. BMC Oral Health. 2025;25(1):766. 40405197 10.1186/s12903-025-06137-5 PMC12096631

