# Supplementary material for: Evaluation of dental practitioners’ knowledge and attitudes regarding drug interactions: a cross-sectional survey
Source: BMC Oral Health. 2026 Feb 10;26:485. doi: 10.1186/s12903-026-07832-7 (PMC12990413; doi:10.1186/s12903-026-07832-7)
Supplement: Supplementary file 1 — Supplementary Material 1. [file 12903_2026_7832_MOESM1_ESM.docx]

# Study Questionnaire

## Demographic Information

1. How many years of professional experience do you have? *

- 0–5 years
 - 6–10 years
 - 15–20 years
 - 20+ years

2. Have you received specialty or PhD education in dentistry? *

- Yes
 - No
 - Currently continuing my education

3. Which dental specialty are you working in? *

- General Dentistry
 - Oral and Maxillofacial Surgery
 - Periodontology
 - Pediatric Dentistry
 - Endodontics
 - Restorative Dentistry
 - Orthodontics
 - Prosthodontics
 - Oral Diagnosis and Radiology

4. What is your working environment? *

- Private Clinic
 - Public Hospital
 - University Hospital
 - Private Hospital
 - Private Practice
 - Other: ______

## General Knowledge Level and Clinical Practices

5. Which drug groups do you think have significant interactions with medications used during dental treatment? *

- Anticoagulants
 - Antibiotics
 - Antidepressants
 - Corticosteroids
 - Beta blockers
 - All of the above
 - None
 - Other: ______

6. In your clinical practice, which drug interactions do you pay most attention to? (Select all that apply) *

- Anticoagulants
 - Antibiotics
 - Corticosteroids
 - Beta blockers
 - All of the above
 - None
 - Other: ______

7. Do you know the mechanisms of action of the drugs you use? *

- Yes
 - No
 - Partially

8. Do you always ask your patients about the medications they use during anamnesis? *

- Yes, in every patient
 - Mostly
 - Sometimes
 - No

9. How do you find out about the medications your patients are using? (Select all that apply) *

- By checking the e-prescription or patient records system
 - By requesting the patient’s medication list
 - Only by asking medical history
 - I mostly do not ask

10. How do you access information about your patients’ medications? *

- My own knowledge base
 - Medical databases (Micromedex, UpToDate, etc.)
 - Consulting colleagues
 - Information from pharmaceutical companies
 - Other: ______

## Experience with Drug Interactions

11. Have you ever encountered a complication due to drug interactions during your professional life? *

- Yes, multiple times
 - Yes
 - No, never
 - Not sure

12. Which of the following antibiotics can inhibit the CYP3A4 enzyme and cause drug interactions? *

- Clarithromycin
 - Azithromycin
 - Metronidazole
 - Tetracycline
 - I don’t know
 - All macrolide antibiotics

13. Which of the following drug groups do you think can interact with macrolides (e.g., erythromycin, clarithromycin, azithromycin) and cause serious side effects? (Select all that apply) *

- Statins
 - Antiarrhythmics (e.g., amiodarone)
 - Benzodiazepines (e.g., midazolam)
 - SSRIs (e.g., citalopram)
 - Calcium channel blockers (e.g., verapamil, diltiazem)
 - Fluoroquinolones (e.g., levofloxacin, moxifloxacin)
 - None
 - I don’t know

14. Colchicine is metabolized by P-glycoprotein and CYP3A4. Which macrolide antibiotics may increase colchicine toxicity risk through these mechanisms?

- Erythromycin
 - Clarithromycin
 - Azithromycin
 - None
 - All

15. Have you ever experienced a drug interaction in a patient with a history of colchicine use? *

- Yes
 - No
 - Not sure

16. Which substance, when used together with metronidazole (commonly prescribed in dentistry), may cause severe nausea, vomiting, and palpitations? *

- Caffeine
 - Alcohol
 - Aspirin
 - Beta blockers
 - I don’t know

17. Which of the following drugs, when used with non-steroidal anti-inflammatory drugs (NSAIDs) such as ibuprofen and diclofenac, may negatively affect kidney function? *

- Beta blockers
 - Antibiotics
 - ACE inhibitors (ramipril, enalapril)
 - SSRIs (fluoxetine, sertraline)
 - I don’t know

18. Which of the following drugs commonly used in dentistry may interact with warfarin and increase INR, thereby raising the risk of bleeding? *

- Clarithromycin
 - Amoxicillin
 - Paracetamol
 - Azithromycin
 - I don’t know

19. Do you know the interactions of drugs commonly prescribed in dentistry with warfarin in patients using warfarin? *

- 1-No knowledge
 - 2-Limited knowledge

-3- Intermediate knowledge

-4 Good knowledge
 - 5 – Advanced knowledge

20. How competent do you feel in managing drug interactions? (Likert scale: 1 = Inadequate, 5 = Very competent) *

- 1 Inadequate
 - 2 Limited
 - 3 Moderately competent
 - 4 Competent
 - 5 Very competent

## Education and Decision Support

21. Which topics related to drug interactions in dentistry would you like to receive more training in? *

- Specific drug interactions
 - Patient safety in drug use
 - Rational drug use and use of digital tools in drug interactions
 - Other: ______

22. If there were a drug decision support system available for dentists when prescribing, would you use it? *

- Yes
 - No
 - Not sure

23. In your opinion, which information source is the most reliable to consult when you need knowledge about drug interactions? *

- E-prescription system
 - Turkish Medicines and Medical Devices Agency (official website)
 - Vademecum
 - My colleagues
 - Other: ______
